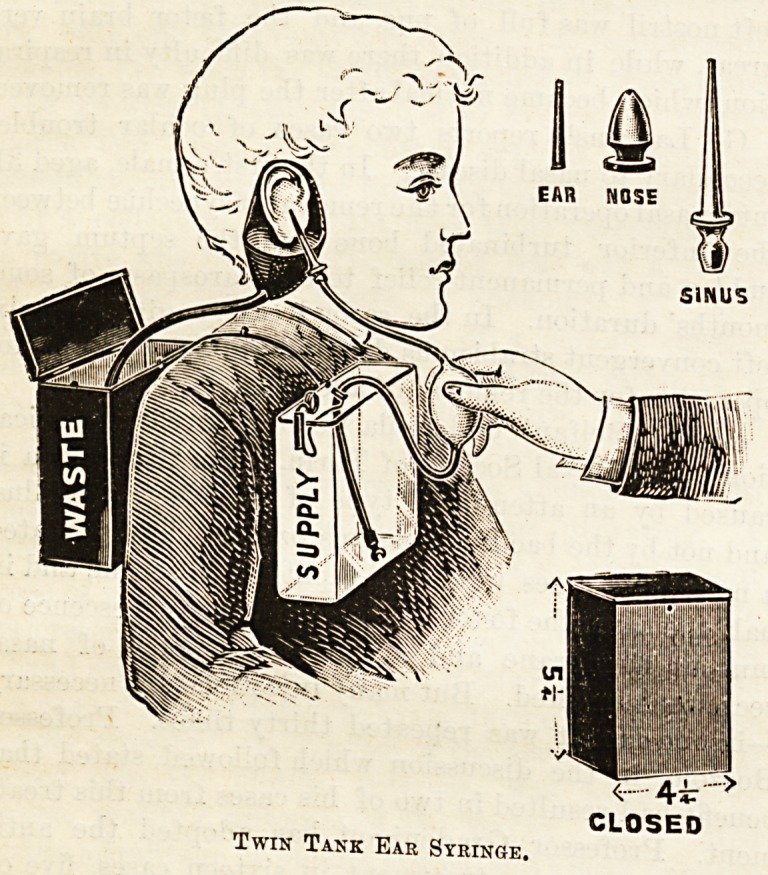# New Appliances and Things Medical

**Published:** 1896-10-10

**Authors:** 


### NEW APPLIANCES AND THINGS MEDICAL.

[We shall be glad to receive, at our Office, 28 & 29, Southampton Street, Strand, London, W.C., from the manufacturers, specimens of all new preparations and appliances which may be brought out from time to time.]

### STERILIZED MILK.

(The Excel Sterilized Milk Company, 28, Victoria Street, S.W.)

All sensible people have their milk boiled as soon as it enters the house; in this way, doubtless, much illness is avoided. But simple boiling, unless repeated, does not render milk germ free, although it goes a long way towards it. The milk which is sterilized by the above company is absolutely germ free, and supplied in hermetically sealed bottles. It is unnecessary to enter into the details of the process by which this result is obtained, but they are such as could not be carried out effectually in a private household. The milk thus rendered germ free will, of course, remain indefinitely without turning sour or suffering coagulation of the casein; the cream, however, collects on the surface, and after the lapse of some time can only with difficulty be properly distributed through the rest of the milk. This is, perhaps, the only objection to sterilized milk of this kind—an objection which can be obviated by not purchasing the milk in too large a quantity at a time. The advantages are many; perhaps the most prolific source of infectious disease is removed. Unturned milk is always at hand—much trouble is saved, and the expense hardly increased at all.

### TWIN TANK EAR SYRINGE.

(Reynolds and Branson, Briggate, Leeds.)

To syringe an ear single handed is often attended with difficulty, and it is not always easy to prevent overflow from the receptacle. On the suggestion of Dr. J. J. Jackson, of East Ardsley, Yorks, Messrs Reynold and Branson have made a praiseworthy attempt at rectifying these

Twin Tank Ear Syringe.

inconveniences. As may be seen from the illustration, a supply and waste tank are slung over the shoulders of the patient, the aural reservoir drains into the latter, and the end of the syringe is clipped and fixed immovably in the former. The operator has, in consequence, his hands free for the mechanical process of injection and the patient can sit in an easy position. The various parts of the apparatus can be conveniently and compactly packed into the metal tank.

### SURGICAL DRESSINGS.

(Robinson and Sons, Limited, Wheat Bridge Mills, Chesterfield.)

We have received samples of two forms of a valuable innovation in surgical dressings from the above firm. The first goes by the name of "Robinson's Tissue," and consists of alternate layers of cotton wool and cellulose wadding. Cellulose wadding has been proved to be one of the most absorbent of all dressings; it has, however, the undesirable property of forming a paste-like mass when saturated, through which discharges do not easily become distributed. Cotton wool, on the other hand, though very absorbent, does not distribute the discharge far from the primary seat of absorption. When, however, these two forms of dressing are successively united, as in the dressing under discussion, the valuable properties of each is seen to the greatest advantage, and we believe that the Robinson tissue will prove very useful in surgical practice. The second preparation goes by the name of "Robinson's Dressing," and is the same as the former, except that it is not arranged between layers of gauze. We may add that we have had an opportunity of testing these dressings practically, and that our own appreciation of them was no greater than was that of the nurse and the patient.

### ALBERT WATER.

(Messrs. Eugene Oppenheimer, 7, Catherine Court, Tower Hill, E.C.)

We have had our attention directed to this new table water, which is being introduced to the public and the medical profession by the above firm. From trial which we have made of it we regard it as quite one of the most agreeable of the table waters at the present time upon the market. It has a slightly saline taste, but although oxide of iron is present in small quantity, it is devoid of the disagreeable taste which characterises most waters which contain this ferruginous element, and we should not have been able to detect its presence by mere taste alone. Potassium, sodium, lithium, magnesium, and manganese salts are all present in small quantity, combined in various proportions with the radicles of carbonic acid; sulphuric, phosphoric, hydrobromic, hydrochloric, iodic, and boracic acid salts of the heavy earths, calcium, barium, and strontium are also present. Among table waters possessing mild alterative and antacid qualities, we believe that Albert Water deserves to secure a recognised position.

### SONIX FLOUR AND CLEMENT'S FOOD.

(Clifton Clement and Co., Burnley.)

Sonix flour is intended for the manufacture of the bread which goes by the same name, and which has already proved itself of considerable value to weak digestive powers. It is claimed for it that from the processes to which the flour is submitted the indigestible and irritating elements are removed without eradicating any of the nourishing and bone-forming constituents. From examination to which we have put it, the flour seems well provided with the phosphatic and other ingredients of the whole grain which are necessary for the maintenance of muscular and cerebral activity.

Clement's Food, which is intended for infants, invalids, and aged persons, is easily prepared, pleasant to take, and readily digested and absorbed. It should certainly be found of use in the cases for which it is recommended.

### FLORADOR FOOD.

(The Florador Food Company, Glasgow.)

This excellent preparation of wheat-flour is in a finely granular condition, in grains of three sizes—either fine, medium, or large, according to the taste of the purchaser. From tests to which we have submitted it, we regard it as a pure preparation of wheat, containing a high percentage of the glutinous or albuminoid contents of the whole wheat. Consequently, as a food and flesh former, it has more valuable qualities than the highly-refined flours and arrowroots which are practically little more than pure starch. For the general purposes of cooking and for young people it is a preparation that we can highly recommend.

### DEE-CEE FLOUR AND HELM COCOA.

(David Challen, Limited, London.)

We have received samples of the above goods from the London agents. The first-named is an American leavened flour, which requires no addition of salt, yeast, or baking powder in the making of bread, pastry, cakes, or other confectionery. It is claimed for it that the method of preparation ensures the preservation of the natural phospatic and nutritious elements of the wheat, which are partly lost or destroyed in the ordinary methods of manufacture. The printed recipes which are supplied with the flour for the preparation of bread, pastry, scones, &c., show considerable simplification over those usually found in standard cooking books, and from a trial to which we have submitted the flour in one or two instances, the statements made in its favour seem perfectly justified.

With regard to the Helm Cocoa, we consider it to be a very good sample of a highly soluble cocoa. The absence of free fat, the excellence of the flavour, and the ease with which it can be prepared, should recommend it to all who appreciate this wholesome beverage.

## Figures and Tables

**Figure f1:**